# Strength in numbers? Grouping, fund allocation and coordination amongst the neglected tropical diseases

**DOI:** 10.7189/jogh.03.020302

**Published:** 2013-12

**Authors:** Anand Bhopal, Thomas Callender, Ailie Flora Knox, Sadie Regmi

**Affiliations:** 1 School of Medicine, University of Manchester, United Kingdom; 2 Institute for Science, Ethics and Innovation, University of Manchester, United Kingdom

Neglected tropical diseases (NTDs) is a term used to describe a heterogeneous group united not by pathophysiology or geography, but by their perpetuating the poverty of “invisible people”. Their burden is laid on one billion of the world’s poorest, who are both at greater risk of contracting the diseases, and of being trapped in poverty by the ensuing effects on their health [1]. The diseases tend to co–exist and can be found in 149 of the 193 countries in the world, of which 100 countries are co–endemic for at least two of the NTDs and 30 countries are endemic for six or more [2].

**Figure:**
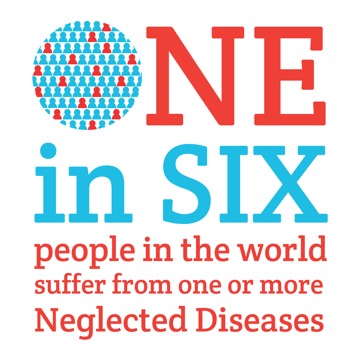
Photo: Graphic designed by Jennifer Matsumoto for UAEM (courtesy of the author)

As use of the term “NTD” has grown in recent years, its success in collecting together a group of diseases that are largely unheard of in high–income countries and using their combined burden to give the whole group added moral, political and economic weight, has been significant. Although precise estimates vary, grouped together the NTDs have a combined global disease burden comparable to that of diseases such as tuberculosis, malaria and human–immunodeficiency virus/acquired immunodeficiency syndrome (HIV/AIDS) [3]. This combined power has brought these diseases from the halls of global health institutions to the attention of a wide range of stakeholders including the media, politicians, philanthropists and the general public. Both scientific interest in the diseases, as measured by research publications, and internet searches for constituent diseases through Google and Yahoo!, have increased over the last decade [4]. Similarly, from 2007 to 2011, the funding for NTDs increased by over 70% [5].

However, the concept of NTDs is not being utilized to its full potential. Lobbying for funding, particularly regarding increasing access to currently available treatment, is still often done on an individual disease basis, and there is no discernible link between indicators such as research and development (R&D) funding and attributable disease burden in DALYs and deaths. Greater global coordination for the diseases, to a degree met by the London Declaration [6], may unravel with competing health issues coming to the fore and the partial completion of the main aims of the declaration. Over the past 18 months great progress has been made towards achieving the goals of the WHO roadmap to NTD control. Yet, as seen with previous control programmes, long–term international support and coordination is needed if gains are to be built upon rather than allowed to slide [7,8]. Although initiatives such as the London Declaration have improved collaboration in this field, they are limited in their scope to truly coordinate the fight against NTDs in the post–2020 era. An international coordinating committee should harness the combined power of these diseases to lobby on their behalf, collecting funds that will then be distributed on a more equitable and transparent basis, whilst ensuring the long–term monitoring and viability of programmes put in place. We aim to expand on the need for an international coordinating committee and attempt to outline the roles of such a committee.

## NTD BURDEN AND R&D FUNDING

**Figure 1:**
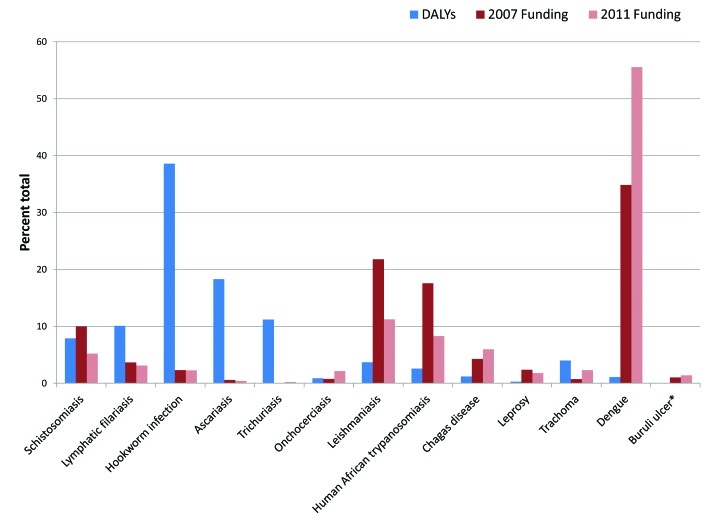
Misalignment of disease burden and funding. Discrepancies in disability adjusted life years (DALY) and funding allocation for various neglected tropical diseases. Estimated DALYs from Hotez et al. [3]; funding data from G–finder 2012 [5]. Asterisk – DALY burden unknown [3].

There is no precise definition as to which diseases constitute NTDs: WHO officially lists 17 diseases [2], the Public Library of Sciences uses a wider list of 37 diseases [9], whilst most often the term refers to a ‘core’ of 13 diseases [1]. This is further complicated by the terms neglected tropical diseases and neglected diseases being used interchangeably in academic literature [10]. Different stakeholders using the same term at any given time to encompass different diseases makes it difficult to set specific targets for control or to lobby for funding for NTDs as a group. Consequently, attention and funding are more aligned with the success of advocacy groups for individual diseases, with heavy reliance on pharmaceutical company donations, than to any objective criteria such as disease burden, attributed deaths or the need for new drugs, diagnostics and vaccines (**Figure 1**). A similar discrepancy was described by Enserink in 2009 [11].

Of the 13 “core” NTDs shown in **Table 1**, 37.1% of 2007–2011 NTD R&D funding was directed towards the kinetoplastids (leishmaniasis, human African trypanosomiasis, and Chagas disease), which together represent 7.5% of the DALYs and 20% of deaths caused by NTDs. In contrast, over the same period, the helminthiases (lymphatic filariasis, schistosomiasis, hookworm infection, ascariasis, trichuriasis, and onchocerciasis) which make up 87% of NTD DALYs and 75% of deaths due to NTDs, received only 18.5% of the funds disbursed. Thus, although funding has increased to NTDs as a block since the grouping took root, overall funding has clearly not been shared equitably amongst the diseases, as illustrated in **Figure 1**. The funding discrepancies outlined can be explained in part by looking at the product development partnerships (PDPs) in place for different diseases and the high prevalence of certain diseases in middle–income countries; the former generally attract charitable funding and the latter affect countries that are increasingly more able to invest into R&D programmes [11,13]. The result has been an ad hoc approach to R&D into these diseases, rather than an approach aligned with need.

**Table 1:** Disability adjusted life years (DALY), deaths and proportional R&D funding for neglected tropical diseases (NTD)

Disease	DALYs* millions	Deaths*	Proportion of NTD DALYs (%)	Proportion of NTD deaths (%)	% of total NTD funding, 2007–11	Change in funding, 2007–11 (%)^¶^
Schistosomiasis	4.5	280 000^†^	7.9	50.7	6	–8.6
Lymphatic filariasis	5.8	0	10.1	0	3	140
Hookworm infection	22.1	65 000	38.6	11.8	2.3	9.6
Ascariasis	10.5	60 000	18.3	10.9	0.5	24
Trichuriasis	6.4	10 000	11.2	1.8	0.2	630
Onchocerciasis	0.5	0	0.9	0	2	390
Helminthiases^c^	49.8	415 000	87	75.2	18.5	57
Leishmaniasis	2.1	51 000	3.7	9.2	15.3	–9.7
Human African trypanosomiasis	1.5	48 000	2.6	8.7	10.2	–17
Chagas disease	0.7	14 000	1.2	2.5	4.6	140
Kinetoplastids^‡^	4.3	113 000	7.5	20.4	37.1	5.3
Leprosy	0.2	6000	0.3	1.1	2.2	32
Trachoma	2.3	0	4	0	1	470
Dengue^§^	0.7	19 000	1.1	3.4	41.1	180
Buruli ulcer	ND	ND	ND		0.9	140
Total	57.3	552 000				
						
HIV/AIDS	84.5	2 000 000				
Malaria	46.5	890 000				
Tuberculosis	34.7	1 400 000				

**ND – Not determined.**

*Figures quoted from Hotez et al., 2006 [3**]. The data in the table are more widely used than other estimates as they take into some degree of long–term chronic disability and thus are believed to be more accurate [**12**].**

**^†^Death estimates for Africa only.**

**^‡^Figures include funding for multiple diseases as well as individual helminthiases and kinetoplastids funding.**

**^§^Estimates from G–FINDER report (2012) [**5**].**

^¶^**All funding figures calculated from information available in the G–FINDER report (2012) [**5**].**

## LONG TERM SUPPORT

The long–term commitments needed for the continued success of the 2020 Roadmap and London Declaration must not be underestimated. WHO’s roadmap impresses the vital need to foster skills and systems in host nations independent of vertically integrated global programmes, so that long–term control of NTDs can be achieved [14]. Without health system capacity building in host nations, scaling down of global efforts and attention can be perilous. In the eight years leading up to 1964, the United Nations Children’s Fund (UNICEF), in conjunction with WHO, undertook to control Yaws, a disease related to syphilis. Their efforts achieved a 95% reduction in cases, from 50 million to 2.5 million, at which point programmes were transferred to local primary health care, without simultaneous attempts to strengthen already overstretched systems [7]. Control of Yaws was lost, and 44 years later WHO had to launch a new elimination attempt [14]. Similarly, in the case of Leprosy, control through drug treatment alone is not enough; education and rehabilitation are also part of the treatment process and must continue even when drug treatment is no longer needed [15]. A ‘post elimination strategy’ is required for the long–term control of the disease as it will inevitably be difficult to generate financial and political support for implementation of appropriate surveillance systems and after care for patients, once the disease has been declared to have been eliminated [16]. The first WHO report on NTDs used the term ‘elimination’ somewhat loosely to refer to the removal of a disease as a public health problem [2]. The second WHO report resolved any ambiguity by defining “elimination” as it is conventionally used, “reduction to zero incidence … in a defined geographical area” [17]. This marks a significant difference in the end goal of the objectives set out in the WHO roadmap to tackle NTDs.

The rhetoric associated with the London Declaration hints at a world free from NTDs post–2020. This raises concerns that NTDs will no longer considered to be sufficiently problematic to draw the support needed for long–term control. A ‘quick fix’ top–down approach is most susceptible to this; strengthening existing health care systems to enable them to deal with NTDs remains vital [8].

## NEW COORDINATING COMMITTEE

We believe the World Health Organisation (WHO) has a central role in overseeing long term control of the NTDs. WHO has a mandate bestowed by member states, which allows the organisation to effectively and accountably coordinate disease control on an international level. The success of the NTD branding tool should perhaps be extended to include a number of other diseases: killers such as diarrhoeal illnesses and pneumonia, both of which have yet to find a branding frame that resonates with the international community, despite their dramatic DALY burden and attributed deaths [18]. Importantly, like other NTDs, these are diseases that perpetuate poverty and controlling them will result in additional downstream economic, humanitarian and developmental benefits [19]. A difficulty with such a WHO–led approach is the stretched finances of the organisation, in light of emerging global health threats, including the burden of non–communicable diseases, which demand ever greater resources.

The WHO Department of Control of Neglected Tropical Diseases has made great strides in NTD control since its establishment in 2005 [17]. In 2013, the first ever World Health Assembly resolution on all 17 NTDs was passed. To date, the department has mostly concerned itself with what were previously termed “tool–ready” diseases and laudably aims to maximise access to NTDs for which we currently have control measures. However, many of the NTDs, including Chagas disease, leishmaniasis and Human African trypanosomiasis, are still in need of innovative solutions [13].

We propose the establishment of a committee within the WHO Department of Control of Neglected Tropical Diseases which primarily concerns itself with two aims. First, the committee would capitalise on the dynamic nature of NTDs, regularly conducting reviews to reflect the current disease climate and to decide which new diseases should fall under the NTD umbrella. In this way, the successful branding technique of the NTDs can be magnified to deal with current and future neglected diseases, and help break the destructive cycle of poverty.

Second, by advocating on behalf of the diseases as a group, the committee could draw on the strengths of the NTD concept and aid the disbursement of resources on a more equitable basis. A remit to focus on long term control would allow the committee to supplement the great strides made under the London Declaration and widen the scope of efforts to consider other NTDs not addressed under the WHO 2020 roadmap. Coordinated resource allocation can jointly tackle the multiple diseases endemic in an area. In addition a degree of “means testing” can be used in order to ensure R&D finances are directed towards the less well–funded NTDs and away from those which are already being successfully tackled. Member states at the sixty–sixth World Health Assembly this year endorsed the establishment of an observatory to monitor global health R&D investments, including investments into NTDs [20,21]. Using this in conjunction with data from recent efforts to measure the global burden of disease will aid stakeholders to achieve better alignment of resource allocation and health needs [22].

Ultimately, individual disease prevention programmes are inherently limited in their scope to tackle health issues as holistically as a multi–disease approach; the latter, by emphasising the multiple factors which cause afflictions, more effectively place people rather than diseases at the centre of efforts. Some NTDs are treated together with the same medicines; additional control of multiple diseases in co–endemic regions can be achieved more cost–effectively with an integrated treatment approach [1,23]. A committee focused on long term control would be well placed to assess the different needs of these populations and harmonise the efforts of the multiple stakeholders working to combat these diseases. The proposed committee would ensure the positive sentiments behind the London Declaration do not fail to achieve their potential due to lack of an obvious source of coordination and long term vision. This would help put in place sustainable programmes, ensuring disease control does not wane once the attention of policy makers and eyes of the world’s media move on.

The WHO 2020 Roadmap, strengthened by the commitment of the signatories of the London Declaration of 2012, guarantees that the 13 “core” NTDs will receive attention for the rest of the decade. Even if the goals of the roadmap are achieved, by 2020 only one NTD will have the potential for resurgence as has happened in the wake of previous disease control and elimination programmes, and many of these diseases will still be in need of better drugs, vaccines and diagnostics.

## CONCLUSION

A coordinated approach is vital to improving the health of the world’s poorest people. The NTDs represent only some of the health problems which afflict the “bottom billion” and compound poverty; addressing the wider determinants of health is an important part of NTD control whilst being fundamental to sustained improvements in global health [24].

In the long term, a WHO NTD department led committee serving an expanded group of neglected diseases of poverty, is required. The remit of a post–elimination strategy should not detract from current commitments to tackle NTDs; it is a method to build upon current gains in order to reduce the future disease burden and strain on health systems. Through better coordination of NTD R&D and control efforts, a truly sustainable mechanism can be created to systematically rid the world of these “ancient companions of poverty” [2].
